# Long non-coding RNA SNHG1 activates HOXA1 expression via sponging miR-193a-5p in breast cancer progression

**DOI:** 10.18632/aging.103123

**Published:** 2020-06-03

**Authors:** Jun Li, Tianyu Zeng, Wei Li, Hao Wu, Chunxiao Sun, Fan Yang, Mengzhu Yang, Ziyi Fu, Yongmei Yin

**Affiliations:** 1 Department of Oncology, The First Affiliated Hospital of Nanjing Medical University, Nanjing 210029, China; 2 Department of Geriatric Oncology, The First Affiliated Hospital of Nanjing Medical University, Nanjing 210029, China; 3 Nanjing Maternal and Child Health Medical Institute, Nanjing Maternal and Child Health Care Hospital, Gynecology and Obstetrics Hospital Affiliated to Nanjing Medical University, Nanjing 210029, China

**Keywords:** SNHG1, miR-193a-5p, HOXA1, breast cancer

## Abstract

Breast cancer is the leading cause of cancer death in women worldwide. Long non-coding RNA small nucleolar RNA host gene 1 (*SNHG1*) has been reported to be involved in human diseases, including cancer. Here, we found that *SNHG1* expression was significantly upregulated in human breast cancer tissues and cell lines. We explored the function of *SNHG1* in breast cancer cells using *in vitro* and *in vivo* experiments and found that *SNHG1* promotes breast cancer metastasis and proliferation. The potential molecular mechanism of *SNHG1* in breast cancer cells may involve *SNHG1* acting as a sponge of *miR-193a-5p* to activate the expression of the oncogene *HOXA1*. In summary, our study reveals a novel *SNHG1*/*miR-193a-5p/HOXA1* competing endogenous RNA regulatory pathway in breast cancer progression and may provide new strategies for breast cancer therapy.

## INTRODUCTION

Breast cancer (BRCA) is one of the most common and malignant cancer types and, worldwide, one in eight to ten women will develop breast cancer [1]. Although breast cancer in the early stages can be cured by surgery accompanied by chemotherapy and radiotherapy, metastasis and recurrence are likely and, thus, breast cancer has a high mortality. Breast cancer can be divided into four subtypes according to the expression levels of certain molecules, including the estrogen receptor (ER), progesterone receptor (PR), and HER2 protein, as well as Ki67, which represents tumor cells proliferation [2]. Triple-negative breast cancer (TNBC) belonging to basal-like subtype is negative for ER, PR, and HER2 expression and accounts for ~15–20% of BRCA cases [3, 4]. TNBC an aggressive and high-grade BRCA that is highly metastatic and is one of the hardest to treat [5]. Therefore, finding efficient diagnostic markers and effective therapeutic targets is essential for TNBC clinical treatment.

Long non-coding RNAs (lncRNAs) are non-coding RNAs of >200 nucleotides in length [6]. A large number of literatures have revealed the biological functions and significance of lncRNAs in various cellular processes. The small nucleolar RNA (snoRNA) host gene 1 (*SNHG1*) is a newly defined lncRNA which is located at chromosome 11q12.3. *SNHG1* is aberrantly expressed in many cancers, including lung cancer [7, 8], colorectal cancer [9, 10], gastric cancer [11], and liver cancer [12], and maybe oncogenic leading to tumorigenesis in these cancer types. However, whether *SNHG1* participates in the initiation and progression of BRCA remains unclear.

Crosstalk between lncRNAs and microRNAs (miRNAs), another class of non-coding RNAs that contain ~22 nucleotides, is explained by the competitive endogenous RNA (ceRNA) hypothesis. It is postulated that the lncRNA can bind to the miRNA-response elements (MREs) and act as a 'sponge' to compete with other RNA transcripts, such as mRNAs that share the same MREs [13]. *HOXA1*, a member of the *HOX* gene family, is an epithelial oncogene in breast cancer development and can induce the aggressive transformation of epithelial cells *in vivo* [14].

Here, we show that *SNHG1* is an oncogenic lncRNA that interacts with *miR-193a-5p* to reduce its repression of the *HOXA1* gene and thus accelerate breast cancer development. Our findings indicate that the *SNHG1* lncRNA may become a potential biomarker and clinical target in breast cancer treatment.

## RESULTS

### SNHG1 expression is up-regulated in breast cancer tissues and cell lines

**Figure 1:**
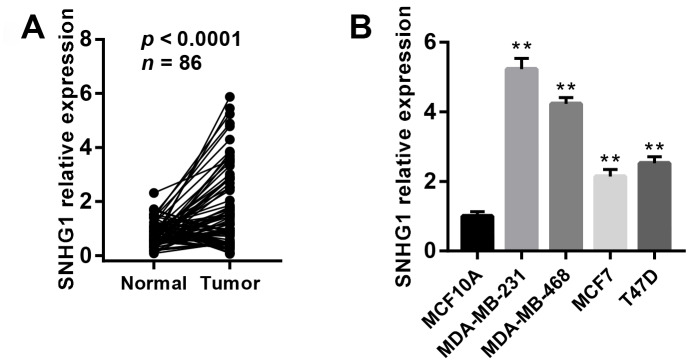
**SNHG1 expression is up-regulated in breast cancer tissues and cell lines.** (**A**) Q-RT-PCR analysis of SNHG1 mRNA levels in collected breast cancer tissues (Tumor) and adjacent normal tissues (Normal). n=86, *p*<0.0001. (**B**) Q-RT-PCR analysis of SNHG1 mRNA levels in MCF10A mammary epithelial cell lines and four different breast cancer cell lines. **p<0.01 compared with MCF10A control.

To explore the function of *SNHG1* in breast cancer progression, we first examined the expression of *SNHG1* in 86 paired breast cancer tissues and adjacent normal tissue using Q-RT-PCR assay. The result suggested *SNHG1* expression was upregulated in 54 out of 86 breast cancer clinical samples (Figure 1A).

We also measured *SNHG1* expression using Q-RT-PCR in MCF10 mammary epithelial cell lines and four breast cancer cell lines (MDA-MB-231, MDA-MB-468, MCF7, and T47D). As expected, *SNHG1* expression was higher in the breast cancer cell lines than in the MCF10A cells (Figure 1B).

### SNHG1 promotes breast cancer cell migration, invasion and proliferation in vitro

**Figure 2:**
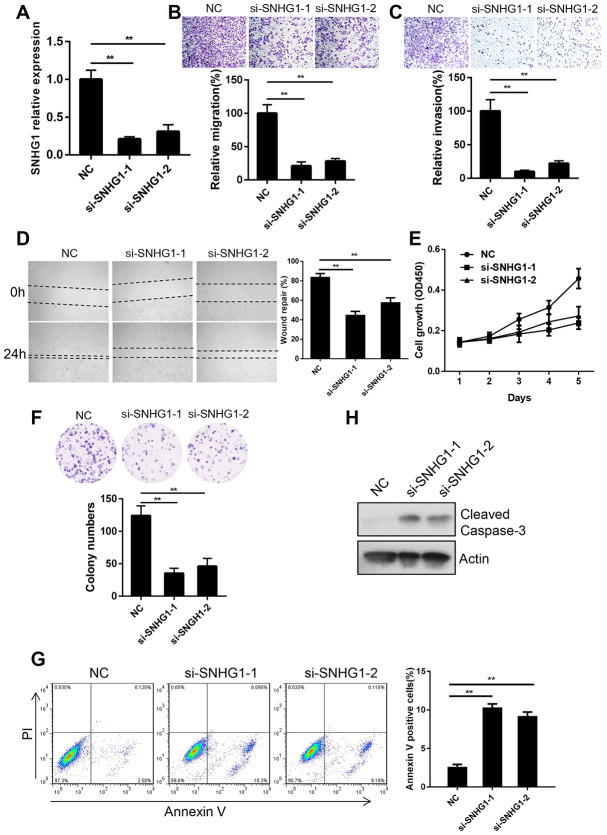
**SNHG1 promotes breast cancer cell migration, invasion and proliferation in vitro.** (**A**) Q-RT-PCR analysis of SNHG1 mRNA levels in MDA-MB-231 cells transfected with NC, si-SNHG1-1 and si-SNHG1-2 siRNAs. **p<0.01 compared with NC control. (**B**, **C**) Migration (**B**) and invasion (**C**) of MDA-MB-231 cells transfected with NC, si-SNHG1-1 and si-SNHG1-2 siRNAs were examined by transwell assay. Representative images of the cells migrated or invaded to the lower chamber side (top panel). Cell migration and invasion capacities were shown as a percentage of NC control (bottom panel). **p<0.01 compared with NC control. (**D**) Representative images of wound healing assay of MDA-MB-231 cells transfected with NC, si-SNHG1-1 and si-SNHG1-2 siRNAs at indicated time (left panel). Wound repair rate was quantified in the histogram (right panel). **p<0.01 compared with NC control. (**E**, **F**) Proliferation of MDA-MB-231 cells transfected with NC, si-SNHG1-1 and si-SNHG1-2 siRNAs was examined by CCK-8 assay (**E**) and colony formation assay (**F**). (**G**) Apoptosis of MDA-MB-231 cells transfected with NC, si-SNHG1-1 and si-SNHG1-2 siRNAs was measured using flow cytometry. **p<0.01 compared with NC control. (**H**) Western blot analysis of cleaved Caspase-3 in MDA-MB-231 cells transfected with NC, si-SNHG1-1 and si-SNHG1-2 siRNAs. Actin was used as loading control.

Since *SNHG1* was most highly expressed in MDA-MB-231 cells, we chose MDA-MB-231 cells to study the function of *SNHG1* in breast cancer progression. We transfected MDA-MB-231 cells with siRNAs targeting *SNHG1* and the interference effect was determined by Q-RT-PCR (Figure 2A). Next, we examined the effect of *SNHG1* downregulation on breast cancer cell migration and invasion by transwell assays. The migratory and invasive capabilities were significantly decreased after *SNHG1* silencing (Figure 2B, 2C). In addition, a wound-healing assay showed that *SNHG1* knockdown led to MDA-MB-231 cells migrating more slowly than control MDA-MB-231 cells (Figure 2D).

Next, we explored the function of *SNHG1* in breast cancer cell proliferation. A CCK-8 cell growth assay showed that silencing of *SNHG1* significantly inhibited the proliferation of MDA-MB-231 cells (Figure 2E). A colony formation assay showed similar results (Figure 2F). We then examined cell apoptosis using cell cytometry and *SNHG1* knockdown enhanced apoptosis of MDA-MB-231 cells (Figure 2G). Cleaved caspase-3, a marker of cell apoptosis, was increased in a Western blot assay following *SNHG1* silencing (Figure 2H).

Together, these data suggest that *SNHG1* plays a role in breast cancer cell migration, invasion, survival, and proliferation.

### SNHG1 promotes breast cancer cell metastasis and proliferation in vivo

**Figure 3:**
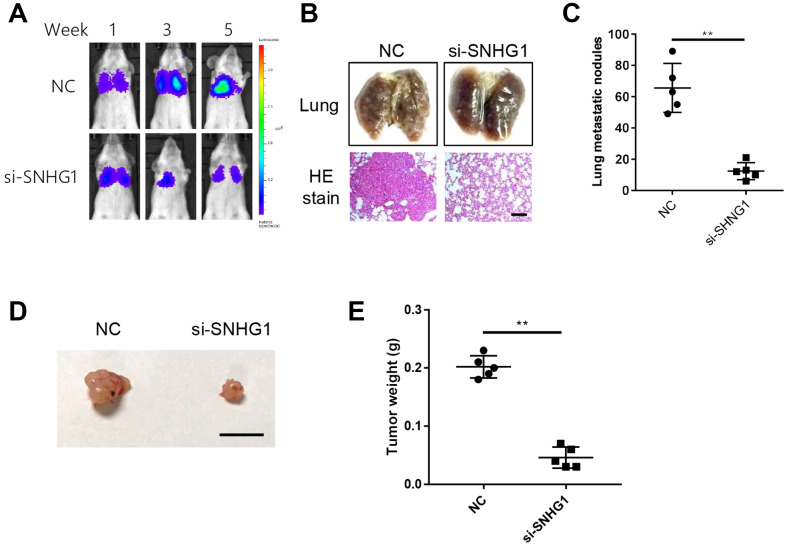
**SNHG1 promotes breast cancer cell metastasis and proliferation in vivo.** (**A**) Representative images of cancer metastasis in SCID mice monitored at indicated time points using in-vivo imaging system. (**B**) Representative images of excised lungs from SCID mice of different groups (top). Representative images of HE staining of lung sections (bottom). Scale bar=400μm. (**C**) Statistics data of lung nodules of SCID mice (n=5 each group). **p<0.01 compared with NC control. (**D**) Representative images of excised tumors from nude mice. Scale bar=1cm. (**E**) The mass of xenograft tumors excised from nude mice (n=5 each group). **p<0.01 compared with NC control.

Next, we examined the effect of *SNHG1* on breast cancer cell metastasis in an *in vivo* mouse model. We injected *SNHG1* knockdown or negative control (NC) MDA-MB-231 cells into severe combined immunodeficiency (SCID) mice via the tail vein. *In vivo* imaging was used to monitor tumor metastasis. The *SNHG1* knockdown group showed significantly reduced tumor growth in the lungs compared with the NC control group (Figure 3A). Similarly, the number of tumor nodules in mice of the *SNHG1* knockdown group was decreased compared with the NC group (Figure 3B, 3C).

We also explored the effect of *SNHG1* on breast cancer cell proliferation *in vivo* using a xenograft mouse model. *SNHG1* knockdown or NC MDA-MB-231 cells were subcutaneously injected into different flanks of nude mice and we found that *SNHG1* silencing led to reduced breast cancer cell growth (Figure 3D, 3E).

### SNHG1 acts as a sponge of miR-193a-5p to activate HOXA1 expression

**Figure 4:**
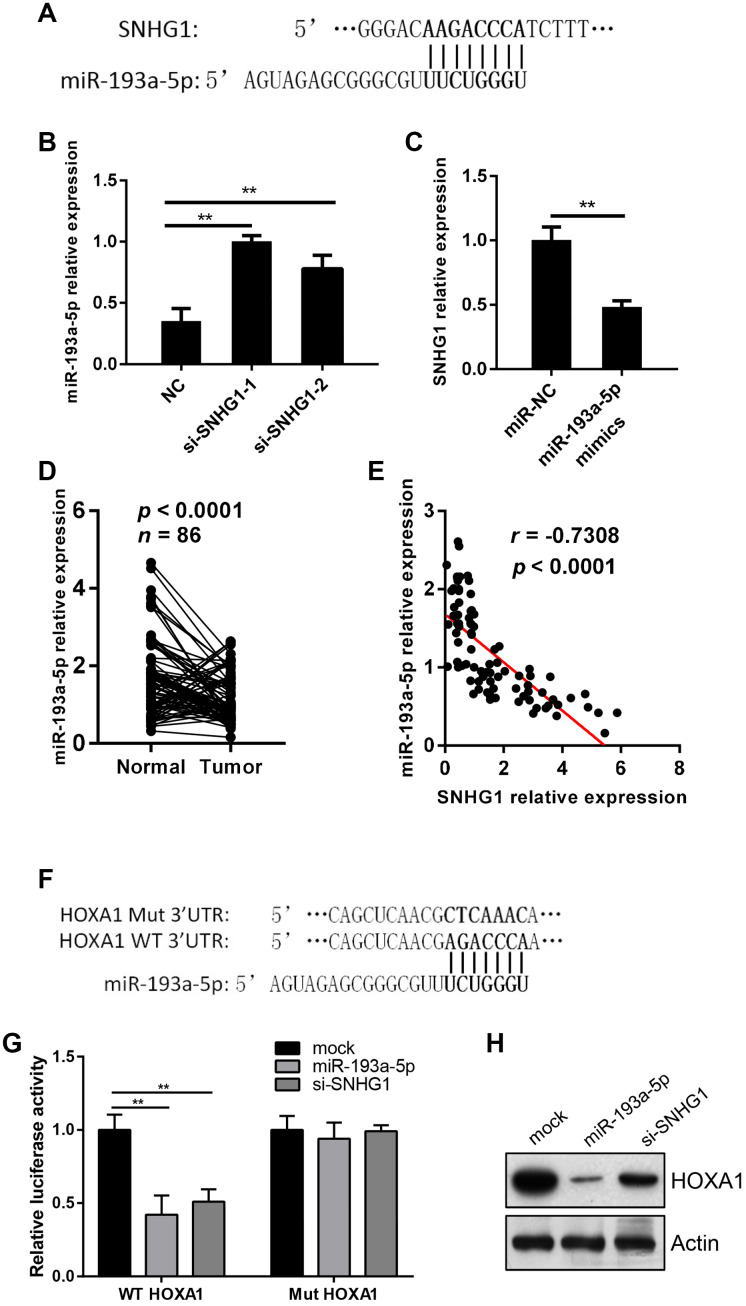
**SNHG1 acts as a sponge of miR-193a-5p to activate HOXA1 expression.** (**A**) Predicated binding sites of miR-193a-5p on SNHG1 gene. (**B**) Q-RT-PCR analysis of miR-193a-5p levels in MDA-MB-231 cells transfected with NC, si-SNHG1-1 and si-SNHG1-2 siRNAs. **p<0.01 compared with NC control. (**C**) Q-RT-PCR analysis of SNHG1 levels in MDA-MB-231 cells transfected with miR-NC and miR-193a-5p mimics. **p<0.01 compared with miR-NC control. (**D**) Q-RT-PCR analysis of miR-193a-5p levels in collected breast cancer tissues (Tumor) and adjacent normal tissues (Normal). n=86, *p*<0.0001. (**E**) Correlation between miR-193a-5p levels and SNHG1 levels in breast cancer tissues. *r*=-0.7308, *p*<0.0001. (**F**) Wild-type (WT) and mutated (Mut) binding sites of miR-193a-5p on HOXA1 3’UTR region. (**G**) Effects of miR-193a-5p and si-SNHG1 on wild-type and mutant HOXA1 3’UTR reporter gene vectors in MDA-MB-231 cells. **p<0.01 compared with mock control. (**H**) Western blot assay of HOXA1 in MDA-MB-231 cells transfected with miR-193a-5p mimcs or si-SNHG1. Actin was used as loading control.

To explore the mechanism of *SNHG1* in breast cancer progression, we used online bioinformatics databases (Starbase and Pictar) to predict the target genes of *SNHG1*. The first 10 miRNA candidates are listed in Table 1. The predicted binding site of *miR-193a-5p* on *SNHG1* is shown in Figure 4A. Silencing of *SNHG1* in MDA-MB-231 cells significantly increased *miR-193a-5p* expression (Figure 4B). Meanwhile, transfection of *miR-193a-5p* mimics inhibited *SNHG1* expression levels in MDA-MB-231 cells (Figure 4C). In addition, we measured *miR-193a-5p* levels in breast cancer clinical samples and found that its expression was relatively lower in tumor tissue compared with paired normal tissue (Figure 4D) and *SNHG1* expression in breast cancer tissues exhibited a significant inverse correlation with *miR-193a-5p* expression (Figure 4E).

**Table 1:** miRNA candidates of SNHG1.

**miRNA name**
hsa-miR-377-3p
hsa-miR-21-5p
hsa-miR-193a-5p
hsa-miR-380-3p
hsa-miR-195-5p
hsa-miR-424-5p
hsa-miR-361-3p
hsa-miR-9-3p
hsa-miR-137
hsa-miR-16-5p

Next, we searched for *miR-193a-5p* targets using the TargetScan and miRDB online databases. We focused on the *HOXA1* gene in this study as is a potential oncogene in breast cancer. The predicted binding site of *miR-193a-5p* on the 3’UTR region of the *HOXA1* gene is shown in Figure 4F. The result of luciferase reporter gene assay showed that *MiR-193a-5p* inhibited the luciferase activity of the wild-type 3’UTR of *HOXA1* but not the mutant one (Figure 4G). Transfection of *miR-193a-5p* mimics into MDA-MB-231 cells significantly suppressed HOXA1 protein levels (Figure 4H). Moreover, silencing of *SNHG1* in MDA-MB-231 cells yielded similar results for the luciferase activity and HOXA1 protein levels (Figure 4G, 4H).

Together, these data suggest that *SNHG1* acts as a sponge to inhibit *miR-193a-5p* and thus activates *HOXA1* expression in breast cancer cells.

### Ectopic expression of HOXA1 reversed the effect of SNHG1 silencing on MDA-MB-231 cells

**Figure 5:**
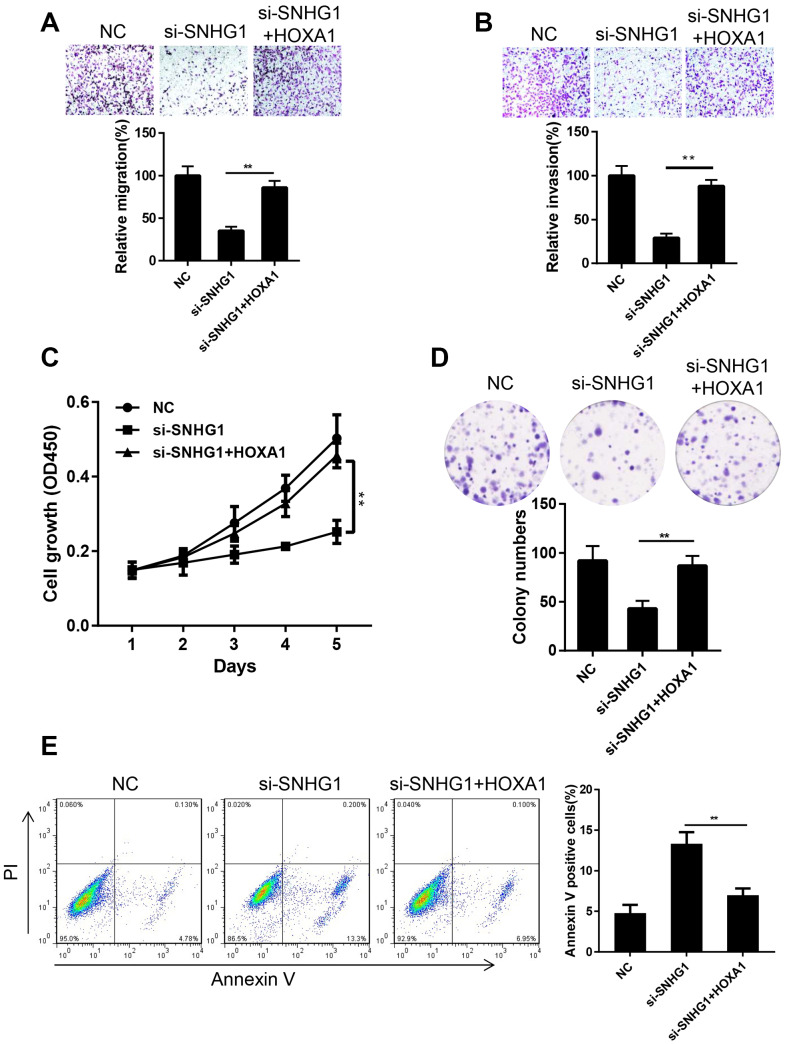
**Ectopic expression of HOXA1 reversed the effect of SNHG1 silencing on MDA-MB-231 cells.** (**A**, **B**) Migration (**A**) and invasion (**B**) of MDA-MB-231 cells transfected with NC, si-SNHG1 and si-SNHG1+HOXA1 were examined by transwell assay. Representative images of the cells migrated or invaded to the lower chamber side (top panel). Cell migration and invasion capacities were shown as a percentage of NC control (bottom panel). **p<0.01 compared with si-SNHG1 group. (**C**, **D**) Proliferation of MDA-MB-231 cells transfected with NC, si-SNHG1 and si-SNHG1+HOXA1 was examined by CCK-8 assay (**C**) and colony formation assay (**D**). (**E**) Apoptosis of MDA-MB-231 cells transfected with NC, si-SNHG1 and si-SNHG1+HOXA1 was measured using flow cytometry. **p<0.01 compared with si-SNHG1 group.

To confirm that *HOXA1* is involved in the *SNHG1* pathway in breast cancer progression, we tested whether enforced HOXA1 expression would eliminate the effect of *SNHG1* silencing in MDA-MB-231 cells. We overexpressed HOXA1 in *SNHG1* knockdown (si-SNHG1+HOXA1) MDA-MB-231 cells. The expression of *SNHG1* and *HOXA1* in NC, si-SNHG1, and si-SNHG1+HOXA1 MDA-MB-231 cells was confirmed using Q-RT-PCR (Supplementary Figure 1A, 1B). Transwell assays showed that *HOXA1* overexpression restored cell migration and invasion capacities (Figure 5A, 5B). In addition, the cell proliferation ability of MDA-MB-231 cells was also reversed by *HOXA1* overexpression (Figure 5C, 5D). Cell cytometry assays indicated that the cell apoptosis rate was reduced after *HOXA1* overexpression in *SNHG1* knockdown MDA-MB-231 cells (Figure 5E). Moreover, we also overexpressed *HOXA1* in MDA-MB-231 cells transfected with *miR-193a-5p* mimics. The transwell assay results suggested that transfection of *miR-193a-5p* mimics inhibits the migration and invasion of MDA-MB-231 cells while *HOXA1* overexpression reverses this effect (Supplementary Figure 2A, 2B). These data above indicate that *HOXA1* is the main target gene of *SNHG1* and *SNHG1/miR-193a-5p/HOXA1* axis plays a critical role in breast cancer progression.

## DISCUSSION

Long non-coding RNAs play a critical role in human diseases, including cancer, and are involved in the regulation of cancer cell growth, migration, invasion, and drug resistance [15–17]. The *SNHG1* gene is located on chromosome 11 and is the host gene of eight snoRNAs. Several studies have revealed that *SNHG1* expression is abnormally elevated in cancer cells and may function as an oncogene to promote cancer progression. For instance, *SNHG1* promotes non-small cell lung cancer progression through inhibiting *miR-101-3p* expression and activating the Wnt/β-catenin signaling pathway [18]. *SNHG1* is also a crucial regulator of colorectal cancer cell proliferation by interacting with *miR-145* [9] and *SNHG1* promotes pancreatic cancer cell growth via regulation of the PI3K/AKT pathway [19]. In addition, a recent study reported that *SNHG1* promoted breast cancer cell growth and invasion by regulating miR-382 [20]. These findings demonstrate that *SNHG1* may act as a potential oncogene in cancer initiation and progression.

In the present study, we found that *SNHG1* expression was significantly upregulated in breast cancer tissues and cells. Silencing of *SNHG1* in breast cancer cells inhibited cell proliferation, migration, and invasion and, thus, *SNHG1* may play an important role in breast cancer development. In addition, we reveal an important target gene of SNHG1 and related molecular mechanism. Although there has been a preliminary report of the function of *SNHG1* in breast cancer [20], our study reveals a novel competing endogenous RNA (ceRNA) axis to elucidate the role of *SNHG1* in breast cancer progression.

The ceRNA hypothesis has been widely used to explain the molecular mechanism of lncRNAs in gene regulation. The hypothesis describes that mRNAs and lncRNAs can talk to each other through a miRNA link. LncRNAs can regulate the expression of mRNAs by competing for shared miRNAs. Several studies have identified the interaction between *SNHG1* and miRNAs. For example, *SNHG1* soaks up *miR-195-5p* to regulate PDCD4 expression in hepatocellular carcinoma [21]. *SNHG1* can also activate the expression of NUAK1 by downregulating *miR-145-5p* and thus induce epithelial-mesenchymal transition in nasopharyngeal carcinoma cells [22]. Here, we found that *SNHG1* could directly interact with *miR-193a-5p* and regulate its expression in breast cancer cells. Moreover, *SNHG1* and *miR-193a-5p* expression levels in breast cancer tissues negatively correlated. These data indicate that *SNHG1* acts as a sponge and soaks up *miR-193a-5p* in breast cancer.

HOXA1, a transcription factor of the HOX protein family, is overexpressed in many tumor cells and is involved in tumorigenesis and the development of several cancers including breast cancer [23, 24]. Here, we found that *HOXA1* was a target of *miR-193a-5p* and its expression is suppressed after transfection of *miR-193a-5p* mimics or *SNHG1* siRNA. Furthermore, *HOXA1* overexpression can eliminate the effect of *SNHG1* silencing in breast cancer cells. These data indicate that the effect of *SNHG1* in breast cancer cells is, at least partially, dependent on HOXA1.

Collectively, our study demonstrates that *SNHG1* promotes breast cancer cell proliferation and metastasis by acting as a sponge of *miR-193a-5p* to activate *HOXA1* expression.

## MATERIALS AND METHODS

### Clinical data

All 86 pairs of BRCA tumor samples and adjacent normal breast tissue samples were collected from The First Affiliated Hospital of Nanjing Medical University and instantly frozen in liquid nitrogen for the following RNA preparation. Related patients provided their signatures to the informed consents of our research. All the procedures were approved by the Ethics Committee of the First Affiliated Hospital of Nanjing Medical University.

### Cell culture

Human normal breast epithelial cell line MCF10A and breast cancer cell lines MDA-MB-231, MDA-MB-468, MCF7, T47D were obtained from the Cell Bank of the Chinese Academy of Sciences (Shanghai, China). All the cell lines were maintained in Dulbecco’s modified Eagle’s medium (DMEM, Gibco Life Technologies, Carlsbad, CA, USA) containing 10% fetal bovine serum (FBS, Hyclone, Logan, UT, USA). Cells were cultured in the incubator at 37 °C provided with 5% CO2 and a humid atmosphere.

### Small RNAs, vectors and transfections

The small interfering RNAs (siRNAs) against SNHG1 or negative control siRNA as well as miRNA mimics were purchased from GenePharma (Shanghai, China). The siRNA sequences were: si-SNHG1-1: 5’-GAUGUACCUUAAAGUGUUA-3’, si-SNHG1-2: 5’-GAGAGGUACUACUAACCAA-3’. The miRNA mimics used were: miR-193a-5p: 5’- UGGGUCUUUGCGGGCGAGAUGA-3’.

The coding sequence of HOXA1 gene were cloned into the BamHI/XhoI sites of pcDNA3 vector. The wild type (WT) and mutant (Mut) sequences of HOXA1 3’UTR region were cloned into the luciferase reporter vector psiCHECK-2 (Promega Corporation, Madison, WI, USA).

All small RNAs and vectors were transfected into breast cancer cell lines using lipofectamine 2000 (Invitrogen, Carlsbad, CA, USA) according to the manufacturer’s instructions.

### RNA extraction and quantitative real-time PCR (Q-RT-PCR)

RNA was isolated from human tissue samples and cell lines using TRIzol Reagent (Invitrogen) following its described methods. Reverse transcriptase PCR was performed using PrimeScript 1^st^ strand cDNA Synthesis Kit (Takara Inc., Dalian, China) to synthesize cDNA from total RNA samples. Subsequently, Q-RT-PCR was performed with FastStart Universal SYBR Green Master (Sigma-Aldrich, St. Louis, MO, USA) and analyzed by a 7500 Fast real-time PCR system (Applied Biosystems, Foster City, CA, USA) under the following program: 95 °C for 10 min followed by 40 cycles of 95 °C for 10 s and 60 °C for 30 s in a total of 20 μl reaction. GAPDH was used to normalize the RNA level as an internal control. All the primers used for Q-RT-PCR were: SNHG1 Forward: 5’-CAGGCATTCAAAGGTTCTGTTAG-3’, SNHG1 Reverse: 5’-GGTACGGCTCCTTTGTTCTCA-3’. GAPDH Forward: 5’-AAGGGCTCATGACCACAGTC-3’, GAPDH Reverse: 5’-ATCACGCCACAGCTTTCCA-3’. For miRNA analysis, cDNA was synthesized with specific stem-loop primers and analyzed using a 7500 Fast real-time PCR system. U6 was used as internal control to normalize the miRNA levels. The primers for miR-193a-5p and U6 were designed and synthesized from GenePharma (Shanghai, China). MiR-193a-5p Forward: 5’- ACACTCCAGCTGGGTGGGTCTTTGCGGGCGAGATGA-3’, miR-193a-5p Reverse: 5’- TGGTGTCGTGGAGTCG-3’. U6 Forward: 5’-CTCGCTTCGGCAGCACA-3’, U6 Reverse: 5’-AACGCTTCACGAATTTGCGT-3’. Relative gene expressions were determined using ΔΔCT method.

### Cell migration, cell invasion, cell proliferation and cell apoptosis analysis

Cell migration was assessed by transwell migration assay and in vitro wound-healing scratch assay. For transwell assay, 1×10^5^ transfected cells resuspended in 100 μl serum-free media were seeded onto the upper chamber of 8 μm transwell (Corning, NY, USA). DMEM supplemented with 10% FBS was added to the lower chamber to drive the cells to migrate through the membrane. To estimate cell invasion, chambers coated with matrigel were used under the same conditions as in transwell migration assay. Cells left on the lower chamber side of the membrane were counted under a microscope (Olympus) with three random fields (100×magnification).

For the scratch assay, cells were prior plated onto 6-well plates and incubated overnight to form a monocellular layer. 200 μl pipette tips were used to create scratches on the layer surface and cells were grown to confluent after another 24-h culturing. Representative images were taken under a microscope at 40× magnification at indicated times. Wound repair rate was quantified by dividing the changes in wound width between 24h and 0h by the wound width at 0h.

Cell counting kit-8 (CCK-8, OBiO, Shanghai, China) was used to measure cell proliferation. 3 × 10^3^ cells were seeded onto each well of 96-well plates in 6 replicates. Cells were cultured for in total 5 days and measured every 24 h using a microplate reader (Thermo Fisher Scientific) at 450nm following the manufacturer’s protocols. Cell colony formation assay was also used to indicate cell growth. Each cell line was plated onto 3 wells of 6-well plates at a density of 500 cells per well and incubated for 2 weeks in the incubator. Cell colonies were then fixed with methanol and stained with 0.1% crystal violet.

Cell apoptosis assay was performed by Annexin-V FITC Apoptosis Detection Kit I (BD Biosciences, San Jose, CA, USA) in accordance to manufacturer’s instructions and analyzed by flow cytometry.

### Protein preparation and western blot

Protein lysates were prepared from transfected cells after 48 h in RIPA lysis buffer (Beyotime, Shanghai, China) and quantified by Pierce™ BCA Protein Assay Kit (Thermo Fisher Scientific). Western blot assay was performed following a standard method. Briefly, even quantities of protein samples were loaded into 10% SDS-PAGEs and transferred onto 0.2 μm polyvinylidene fluoride (PVDF) membranes (Sigma-Aldrich) after electrophoresis. The membranes were blocked with 5% skim milk solved in PBS plus 0.1% Tween-20 (PBST) for 1 h at room temperature and then incubated with corresponding primary antibodies overnight at 4 °C. In the next day, the membranes were washed with PBS-T buffer for 4 times followed by incubation with HRP-conjugated secondary antibodies for 1 h at room temperature. After washing off the secondary antibodies, protein bands were detected using the High-sig ECL Western Blotting Substrate (Tanon, Shanghai, China). β-Actin was served as the internal control and antibodies used in our study were as follows: cleaved Caspase-3 (Abcam, ab2302), HOXA1 (Abclonal, A6924), β-Actin (Abclonal, AC026).

### Animal model

Female SCID and BALB/c nude mice were obtained from the Model Animal Research Center of Nanjing University (Nanjing, China). For tumor metastasis assay, 2×10^6^ MDA-MB-231 cells transfected with NC siRNA or SNHG1 siRNA were suspended in 100 μl DMEM and intravenously injected in SCID mice. Tumor growth were monitored using an in vivo imaging instruments (IVIS Lumina XR, PerkinElmer) at indicated time points. All mice were euthanized at the fifth week and lungs were excised for HE staining. For tumor growth assay, 5×10^6^ MDA-MB-231 cells transfected with NC siRNA or SNHG1 siRNA were suspended in 100 μl DMEM/matrigel (9:1) and subcutaneously injected into nude mice. All animals were euthanized 1 month after injection and tumors were collected for following analysis. All animal experiments were performed following the National Institutes of Health Guide for the Care and Use of Laboratory Animals. Experimental procedures were approved by Institutional Review Board of the First Affiliated Hospital of Nanjing Medical University.

### Dual-luciferase reporter assay

MiRNA mimics and psiCHECK-2 vectors containing WT or Mut 3’UTR of HOXA1 were co-transfected into cells as described before. After 48 h incubation, luciferase activities were measured by Dual-Luciferase Reporter Assay System (Promega) following the manufacturer’s instructions. The intracellular Renilla luminescence represented the expression levels of luciferase gene affected by miRNA mimics and measurements of firefly luciferase were used to normalize Renilla luciferase expression.

### Statistical analysis

All experiments were performed in triplicates and data were displayed as mean ± S.D. The results were analyzed using GraphPad Prism 7.02 (GraphPad Software, La Jolla, CA, USA). The significance of differences between two groups or among multiple groups were assessed using Student's t-test or one-way analysis. P value < 0.05 was considered to be statistically significant.

## Supplementary Material

Supplementary Figures
