# Supplementary material for: Long non-coding RNA SNHG1 activates HOXA1 expression via sponging miR-193a-5p in breast cancer progression
Source: Aging (Albany NY). 2020 Jun 3;12(11):10223–34. doi: 10.18632/aging.103123 (PMC7346023; doi:10.18632/aging.103123)
Supplement: Supplementary Figures [file aging-12-103123-s001..pdf]

SUPPLEMENTARY FIGURES

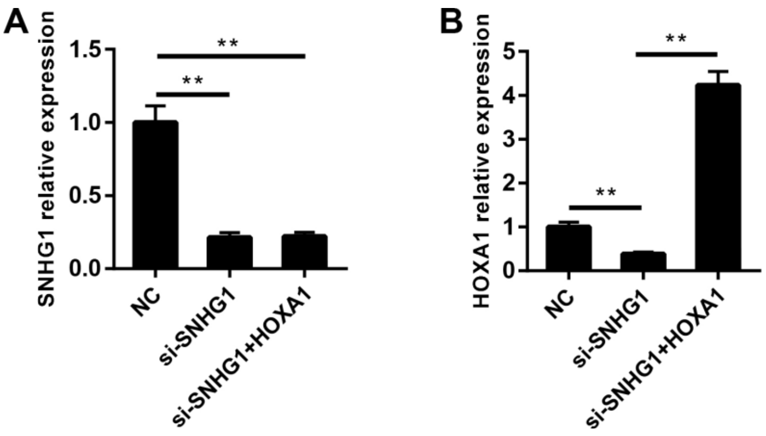

Supplementary Figure 1. SNHG1 and HOXA1 expression in NC, si-SNHG1 and si-SNHG1+HOXA1 cells. (**A**, **B**) The expression of SNHG1 (**A**) and HOXA1 (**B**) in MDA-MB-231 cells transfected with NC, si-SNHG1 and si-SNHG1+HOXA1 were examined using Q-RT-PCR. **p<0.01 compared with indicated control.

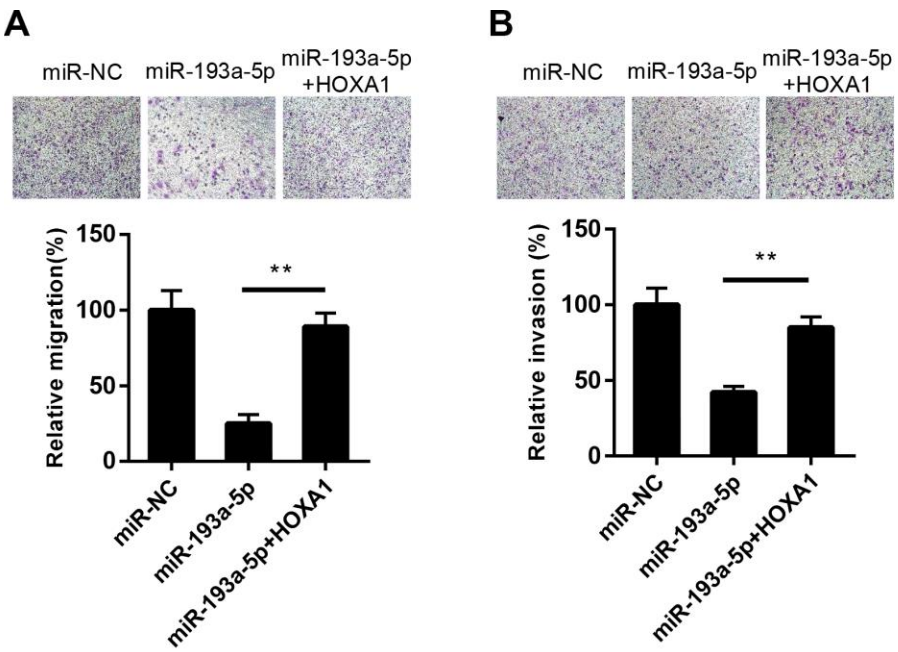

Supplementary Figure 2. Ectopic expression of HOXA1 reversed migration and invasion capacities of miR-193a-5p transfected MDA-MB-231 cells. (**A**, **B**) Migration (**A**) and invasion (**B**) of MDA-MB-231 cells transfected with NC, miR-193a-5p and miR-193a-5p+HOXA1 were examined by transwell assay. Representative images of the cells migrated or invaded to the lower chamber side (top panel). Cell migration and invasion capacities were shown as a percentage of miR-NC control (bottom panel). **p<0.01 compared with miR-193a-5p transfection group.
